# Clinical effects of ursodeoxycholic acid in COVID-19 infection: a systematic review and dose–response meta-analysis

**DOI:** 10.3389/fphar.2026.1719144

**Published:** 2026-04-01

**Authors:** Joo Hye Song, Sung Ryul Shim, Jieun Shin, Won Hyeok Choe, Jiho Park, Tae Hee Lee, Seonghui Kang, Taeho Greg Rhee, Kyu Chan Huh

**Affiliations:** 1 Division of Gastroenterology, Department of Internal Medicine, Konkuk University Medical Center, Konkuk University School of Medicine, Seoul, Republic of Korea; 2 Department of Biomedical Informatics, College of Medicine, Konyang University, Daejeon, Republic of Korea; 3 Evidence Based Research Center, Konyang University Hospital, Daejeon, Republic of Korea; 4 Division of Infectious Diseases, Department of Internal Medicine, Konkuk University of Medical Center, Konkuk University School of Medicine, Seoul, Republic of Korea; 5 Division of Gastroenterology, Department of Internal Medicine, Konyang University College of Medicine, Daejeon, Republic of Korea; 6 Division of Infectious Diseases, Department of Internal Medicine, Konyang University College of Medicine, Daejeon, Republic of Korea; 7 Department of Psychiatry, Yale University School of Medicine, New Haven, CT, United States; 8 Department of Public Health Sciences, University of Connecticut School of Medicine, Farmington, CT, United States

**Keywords:** COVID-19, dose response, infection, meta-analysis, ursodeoxycholic acid

## Abstract

**Objectives:**

Previous studies have shown that ursodeoxycholic acid (UDCA) reduces COVID-19 infection by inhibiting farnesoid X receptor activity, a direct regulator of ACE2. Even though UDCA, an easily accessible medication with few side effects, could be considered for administration to prevent infection and relieve symptoms for COVID-19 infection, there are limited supporting studies with a high-level of evidence and recommendations for the exact dosage of UDCA. We conducted a systematic review and dose–response meta-analysis to evaluate the clinical effect of UDCA in COVID-19 infection.

**Methods:**

Studies were identified through a literature search: PubMed, Embase, and Cochrane from inception to March 2025. We included research related to COVID-19 infection and UDCA. Primary outcomes were COVID-19 infection rate, mortality rate, COVID-19 severe infection risk, ventilator use, hospitalization, ICU hospitalization, and recovery time between UDCA group and controls. The secondary outcome was UDCA dose–response association regarding infection risk. We analyzed for odds ratios (ORs), including infection rate, mortality rate, severe infection risk, ventilator use, hospitalization, and intensive care unit hospitalization, and for standardized mean difference (SMD), including recovery time between UDCA groups and controls. Risk of Bias in Non-randomized Studies of Interventions (ROBINS-I) was used to evaluate bias risk.

**Results:**

Of 188 articles, 15 cohort studies with 716,310 participants (control = 495,276; UDCA treatment = 221,034) were included. The level of risk of bias was seven studies at low, four at moderate, and four at serious. UDCA showed association with a lower risk of infection (OR, 0.69; 95% CI, 0.55–0.86), lower severe infection risk (OR, 0.75; 95% CI, 0.64–0.89), and ventilator use (OR, 0.75; 95% CI, 0.62–0.90) compared to controls.

**Conclusion:**

The findings support evidence for the clinical effects of UDCA for COVID-19 infection. There is a need for randomized trials to evaluate UDCA as a potential prophylactic agent against COVID-19.

**Systematic Review Registration:**

https://www.crd.york.ac.uk/PROSPERO/view/CRD420251019195, identifier #CRD420251019195.

## Introduction

Severe acute respiratory syndrome coronavirus 2 (SARS-CoV-2) is a single-stranded RNA beta coronavirus that causes coronavirus disease (COVID-19) (Coronaviridae Study Group of the International Committee on Taxonomy of Viruses, 2020). The introduction of therapeutic drugs, vaccines, and monoclonal antibodies has significantly improved the management of COVID-19 since the beginning of the pandemic (Kawano-Dourado and Rochwerg, 2021). There are still significant global health concerns despite the transformational impact of vaccines in populations that can access them. High infection rates and significant worldwide mortality are linked to newly emerging SARS-CoV-2 variants (Staller et al., 2021; Harvey et al., 2021; O’Toole et al., 2022). Although monoclonal antibodies are the only prophylactic agents for high-risk groups, effectiveness is limited by the viral spike to evade neutralization (Cao et al., 2022). Moreover, these agents could cause side effects, ranging from mild to serious reactions (e.g., headache, low blood pressure, and allergic reactions).

Preventing SARS-CoV-2 infection by modulating viral host receptors, such as angiotensin-converting enzyme 2 (ACE2), could represent a new chemoprophylactic approach for COVID-19 that complements vaccination (Gaziano et al., 2021; Bartoszko et al., 2021; Mittal et al., 2020). Brevini et al. (2023) identified farnesoid X receptor (FXR), a ubiquitous bile-acid-sensing protein, as a direct regulator of ACE2 transcription in several tissues affected by COVID-19, including the gastrointestinal and respiratory systems. They revealed that ursodeoxycholic acid (UDCA) reduced SARS-CoV-2 infection by suppressing FXR activity. UDCA was most widely used for treating cholestatic hepatopathies and had safety profiles with fewer drug–drug interactions (Shi et al., 2006; Lykavieris et al., 2021; Huang et al., 2023; Stokes et al., 2014; Walker et al., 2020). Using a retrospective registry, Brevini et al. (2023) found a link between UDCA and favorable clinical outcomes after COVID-19 infection. Thereafter, multiple retrospective/prospective observational studies were performed to compare infection rate and the severity of COVID-19 infection between UDCA and controls. However, the characteristics of enrolled patients were heterogeneous, and primary outcomes were various. Moreover, the dose and duration of UDCA which could directly affect preventive effect were very diverse in each study.

In summary, even though UDCA, an easily accessible medication with fewer side effects, could be considered to prevent infection and relieve symptoms for COVID-19 infection, there are limited studies with a high level of evidence and recommendation for an exact dosage of UDCA to support it. Therefore, we conducted a systematic review and dose–response meta-analysis to evaluate the clinical effects of UDCA in COVID-19 infection.

## Methods

This systematic review and meta-analysis was registered in the PROSPERO database (#CRD420251019195). It also thoroughly followed Preferred Reporting Items for Systematic Reviews and Meta-Analyses (Moher et al., 2009) and Meta-analysis of Observational Studies in Epidemiology reporting guidelines (Stroup et al., 2000).

### Data sources and literature searches

The literature search was conducted in the following databases: PubMed, Embase, and Cochrane from inception to 30 March 2025. A literature search strategy was established using Medical Subject Headings terms or Emtree terms suitable for the databases and general text keywords (Supplementary Table 1). The subject headings and text keywords included those related to the intervention of UDCA, comparison groups (i.e., no treatment of UDCA), and outcomes of treatments (i.e., COVID-19 or SARS-CoV-2 infection rate, mortality rate, COVID-19 severe infection risk; following the definition of individual study, ventilator use, recovery time, hospitalization, and intensive care unit (ICU) hospitalization, recovery time).

In this systematic review, there were no restrictions on research design and language, but conference abstracts, editorials, reviews, and commentaries were excluded. Two independent researchers (JH Song and SR Shim) conducted a literature search based on a valid search strategy, and a clinical trial registration site (National Institutes of Health: clinicaltrials.gov) was also manually checked to increase the sensitivity of additional searches.

This study was exempted by the Institutional Review Board at Konyang and Konkuk University, as we utilized publicly available, anonymized data, satisfying the requirements for exemption as detailed in the Department of Health and Human Services regulations.

### Study selection

The criteria for study selection were as follows: 1) population: non-specified; 2) intervention: UDCA; 3) control: no treatment of UDCA. The primary outcomes were COVID-19 infection rate, mortality rate, COVID-19 severe infection risk, ventilator use, hospitalization, ICU hospitalization, and recovery time between UDCA group and controls. The secondary outcome was UDCA dose–response association regarding infection risk. The diagnosis of COVID-19 or SARS-CoV-2 infection used standardized diagnostic criteria (e.g., rapid antigen diagnostic tests, nucleic acid amplification tests, polymerase chain reaction, and/or International Classification of Diseases, 10th Revision). To establish the integrity of the research collected, cross-checking was conducted after independent review by multiple researchers. If there was a disagreement between researchers in the literature collection process, it was resolved through a meeting of all researchers, and the final collected literature was selected after agreement among the entire research group.

### Data extraction

Basic details about the studies (first author, year of publication, country, and study design), patient characteristics (age, female rate, number of patients, COVID-19 vaccination rate, and COVID-19 diagnostic criteria), treatment and controls (intervention and control, dosage, and duration of UDCA), and outcome variables (COVID-19 infection rate, mortality rate, COVID-19 severe infection risk, ventilator use, hospitalization, ICU hospitalization, and recovery time) were extracted from the articles included using a pre-defined data extraction form. For data integrity, studies with unclear outcomes or missing data were excluded from the analysis. To avoid overlapping datasets, national databases were used if the same study presented a single-center cohort and a national database.

When extracting data for dose–response analysis, we used the median of the dose groups. However, if the upper and lower limits of the dose groups were open, the range of the median of the immediately preceding interval was subtracted or added (Shim and Lee, 2019). For example, Lee et al. (2024) was calculated as 150 mg and 450 mg, respectively, corresponding to the median for two groups with dose ranges of less than 300 mg and greater than 300 mg. Hu et al. (2024) used the risk of the entire dose (over 5 mg/kg/day) when analyzing overall effect of UDCA, and the risk of individual dose groups from 300 to 1,050 mg when performing dose–response association analysis. If there was no information about exact daily dose in an individual study, we calculated it using average body weight (Alert, 2005; Committee, 2012). Colapietro et al. (2023), John et al. (2023a), Liu and Wang (2023), Li D. et al. (2024), and Hu et al. (2024) calculated daily dose using average body weight.

### Statistical analysis

We analyzed for odds ratios (ORs), including infection rate, mortality rate, severe infection risk, ventilator use, hospitalization, and ICU hospitalization, and for standardized mean difference (SMD), including recovery time between UDCA groups and controls**.** Statistical heterogeneity was evaluated using Cochran’s *Q* test and Higgins’ *I* ^2^ statistic. The random effects model was used when *I* ^2^ was greater than 50%, and the fixed effects model was used when it was less than 50% (Shim and Kim, 2019). The restricted maximum likelihood (REML) estimator was used to obtain the pooled overall effect sizes and 95% CIs for the outcomes (Veroniki et al., 2016).

Each moderator was subjected to meta-regression analysis for continuous variables and meta-analysis for categorical variables. An REML estimator was used to estimate the variance of the true effects to analyze potential moderators (Shim and Kim, 2019).

To evaluate the risk according to the UDCA dose, regression analysis was conducted by collecting the risk by dose of individual study results (Shim and Lee, 2019). First, distribution of dose and risk was examined through a scatterplot, and then a regression coefficient was calculated to clearly visualize the linear relationship between two variables. To help understand the risk according to the regression coefficient, it was analyzed through index transformation of regression coefficients by specific dose. A two-sided *p-value of* ≤ 0.05 was considered statistically significant. All analyses were conducted using R software version 4.3.1 (R Foundation for Statistical Computing).

### Assessment of potential publication bias

Publication bias was analyzed using a funnel plot. This was a schematic diagram of the ORs, and standard error of infection rate, mortality rate, severe infection risk, ventilator use, hospitalization, ICU hospitalization, and recovery time. If there was no publication bias, individual studies were symmetrically distributed at the top of the funnel, whereas if there was a publication bias, they were relatively distributed outside the funnel if they showed asymmetry. In addition, summary statistics of publication bias were tested using the Egger linear regression test and Begg and Mazumdar rank correlation tests (Shim and Kim, 2019; Egger and Smith, 1998; Begg and Mazumdar, 1994).

### Quality assessment

Risk of Bias in Non-randomized Studies of Interventions (ROBINS-I) is a tool to evaluate the risk of bias for non-RCT studies (Sterne et al., 2016). The entire evaluation domain consists of three major parts (before, during, and after the intervention) and consists of seven items—pre-intervention: 1. bias due to confounding, 2. bias in selection of participants into the study; during intervention: 3. bias in classification of interventions; post-intervention: 4. bias due to deviations from intended interventions, 5. bias due to missing data, 6. bias in measurement of outcomes, and 7. bias in selection of the reported results. The quality of the evidence related to the estimation of benefits and risks was displayed according to specific conditions.

## Results

### Study selection

**FIGURE 1:**
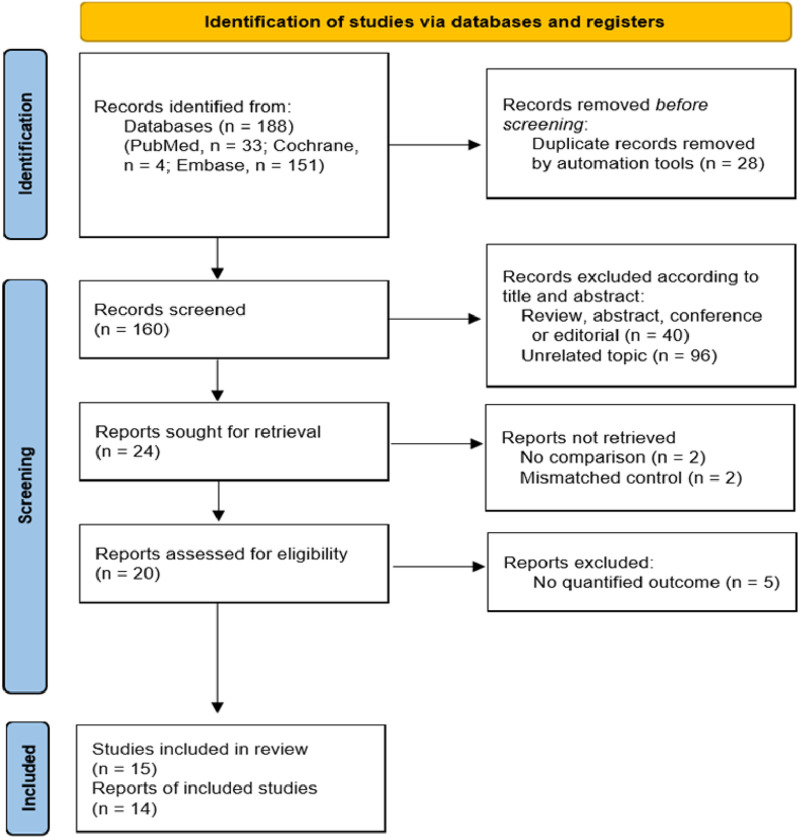
PRISMA (Preferred Reporting Items for Systematic Review and Meta-Analyses) study selection flowchart.

In the initial literature search, 188 articles were searched through a number of electronic databases (PubMed, n = 33; Cochrane, n = 4; and Embase, n = 151). Of these, 28 were automatically removed through the literature reference management program. The remaining 160 articles were additionally removed by checking the title and abstract. We selected 24 articles for full-text screening, and 15 were finally selected, excluding two without a comparison group, two with control matching errors, and five without a valid outcome measure. This final 15 was evaluated for quality assessment, and 14 were subjected to quantitative synthesis meta-analysis, except for Hu’s study, where the outcome is only one asymptomatic infection rate (Figure 1) (Lee et al., 2024; Hu et al., 2024; Okushin et al., 2025; Hu et al., 2025; Yu et al., 2024; Okushin et al., 2024; Moon et al., 2024; Marrone et al., 2024; Li M. et al., 2024; Li D. et al., 2024; Corpechot et al., 2024; Liu and Wang, 2023; Li et al., 2023; John et al., 2023a; Colapietro et al., 2023).

A systematic review and meta-analysis of the 15 studies involving 716,310 patients (control, n = 495,276 vs. UDCA treatment, n = 221,034) was conducted to assess the detailed differences and subject descriptions provided in Table 1 and Supplementary Table 2. The studies were conducted in Western (France, Italy, and USA) and Eastern (China, Korea, and Japan) countries. The mean age ranged from 50.0 to 74.0 years (except for Liu and Wang, 2023), and the proportion of female patients ranged from 25.1% to 83.1%. All were observational studies, and eight were analyzed as pseudo-randomized effects by adjusting the balance of covariates at baseline using propensity score matching (PSM).

**TABLE 1:** Characteristics of included studies.

Study	Age	Female rate	Country	Study design	Population
Hu et al. (2025) ^a^	62	71.52% UDCA; 71.52% non-UDCA	China	Retrospective case–control, single-center by age and sex matching (7 December 2022 to 23 January 2023)	COVID-19 in patients with chronic liver diseases (309 for UDCA; 309 for non-UDCA)
Okushin et al. (2025)	73	45.8% UDCA; 45.5% non-UDCA	Japan	Retrospective case–control by PSM (February 2020 to December 2022)	COVID-19 in patients with chronic hepatitis B or C (579 for UDCA; 5,834 for non-UDCA)
Corpechot et al. (2024)	56.7 for UDCA; 55.0 for non-UDCA	71.7% UDCA; 35.9% non-UDCA	France	Retrospective cohort, multi-center (1 March 2020 to 31 December 2020)	PBC and PSC for UDCA vs. CHB and CHC for non-UDCA (1,322 for UDCA; 8,825 for non-UDCA)
Hu et al. (2024) ^b^	50	25.1 UDCA; 17.2% non-UDCA	China	Retrospective cohort, multi-center by PSM (January 2015 to December 2022)	Immunosuppressed recipients for liver transplantation (326 for UDCA; 571 for non-UDCA)
Lee et al. (2024) ^c^	70 for UDCA; 40 for non-UDCA	36.3% UDCA; 35.3% non-UDCA	Korea	Retrospective cohort study using NHIS by PSM (from 2015 to 2021)	Nationwide cohort (220,005 for UDCA; 4,122,560 for non-UDCA)
Li D et al. (2024)	56	63.8% UDCA (73 matched UDCA; 36 for non-UDCA)	China	Retrospective cohort, single-center by PSM (July 2022 to December 2022)	Outpatient (1,040 for UDCA; 64 for non-UDCA)
Li M et al. (2024)	57.94	83.14% UDCA; 68.75% non-UDCA	China	Prospective study with investigative telephone survey (January 2022 to January 2023)	Autoimmune liver disease patients (PBC, AIH, and PSC) (706 for UDCA; 432 for non-UDCA)
Marrone et al. (2024)	69	41.0% UDCA; 40.9% non-UDCA	Italy	Retrospective case–control, single-center by PSM (March 2020 to December 2022)	COVID-19 in patients admitted to emergency department (109 for UDCA; 6,335 for non-UDCA)
Moon et al. (2024)	56.6	47.0% UDCA; 47.3% non-UDCA	Korea	Retrospective cohort study using NHIS by PSM (January 2020 to December 2021)	Chronic liver disease (8,834 for UDCA; 65,240 for non-UDCA)
Yu et al. (2024)	23	38.5% UDCA; 36% non-UDCA	China	Retrospective case–control, single-center (December 2022) with meta-analysis	COVID-19 in patients with no comorbidities (65 for UDCA; 50 for non-UDCA)
Colapietro et al. (2023)	74	45.6% UDCA; 39.4% non-UDCA	Italy	Retrospective case–control, single-center (January 2020 to January 2023)	COVID-19 in patients with cholelithiasis, cholestatic liver diseases, and bone marrow diseases (57 for UDCA; 3790 for non-UDCA)
John et al. (2023a)	62	7.1% UDCA; 5.4% non-UDCA	USA	Retrospective cohort by PSM (January 2008 to December 2018, with follow-up until 11 February 2022)	Cirrhosis (88 for UDCA; 6,523 for non-UDCA)
Li et al. (2023)	53.3	59.6% UDCA; 56.9% non-UDCA	China	Retrospective cohort, single-center by PSM (January 2022 to December 2022)	Patients (hepatitis B and autoimmune hepatitis) (329 for UDCA; 325 for non-UDCA)
Liu and Wang (2023)	4.7 for UDCA; 6.7 for non-UDCA	41.1% UDCA; 43.75% non-UDCA	China	Questionnaire-based study (December 2022 to January 2023)	Children from families and members (146 for UDCA; 80 for non-UDCA). Children’s hospital of Fudan University for liver disease in preceding 5 years
Okushin et al. (2024)	69	69.0% UDCA; 45.1% non-UDCA	Japan	Retrospective case–control, single-center by PSM	Outpatients. No complication information (55 for UDCA; 335 for non-UDCA)

More detail characteristics (intervention, control group, outcome, UDCA treatment duration, COVID-19 vaccination, and COVID-19 diagnosis criteria) shown in Supplementary Table 2.

^a^ Hu et al. (2025) was used only in the quality assessment.

^b^ Hu et al. (2024) used the risk of the entire dose when analyzing individual outcome and the risk of individual dose groups from 300 to 1,050 mg when performing DRMA.

^c^ Lee et al. (2024) was calculated as 150 mg and 450 mg, corresponding to the median for two groups with dose ranges of less than 300 mg and greater than 300 mg, respectively.

UDCA, ursodeoxycholic acid; PSM, propensity score matching; PBC, primary biliary cholangitis; PSC, primary sclerosing cholangitis; CHB, chronic hepatitis B; CHC, chronic hepatitis C; ICU, intensive care unit; ICD-10, International Statistical Classification of Diseases and Related Health Problems, 10th Revision; NHIS, National Health Insurance Service; AIH, autoimmune hepatitis.

### Outcome findings

**FIGURE 2:**
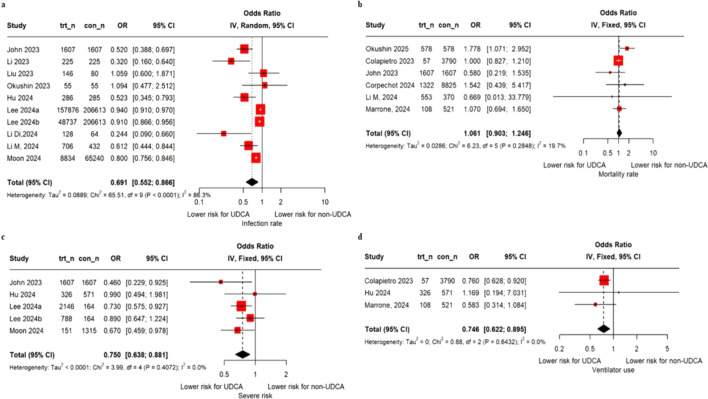
Forest plots for **(a)** infection rate and clinical effect, **(b)** mortality rate, **(c)** severe risk, and **(d)** ventilator use. ^†^ Lee et al. (2024) was calculated as 150 mg and 450 mg, respectively, corresponding to the median for two groups with dose ranges of less than 300 mg and greater than 300 mg; severe risk following the definition of individual study; John et al. (2023a) and Hu et al. (2024) based on NIH COVID-19 severity scale: severe illness: individuals who have an SpO_2_ <94% on room air at sea level, ratio of arterial partial pressure of oxygen to fraction of inspired oxygen (PaO_2_/FiO_2_) <300 mmHg, respiratory rate >30 breaths/min, or lung infiltrates >50%, critical illness: individuals with respiratory failure, septic shock, or multiple organ dysfunction; Lee et al. (2024), one of the following conditions occurred: (1) admission to an intensive care unit, (2) extracorporeal membrane oxygenation treatment, (3) use of a mechanical ventilator, or (4) death; and Moon et al. (2024), complications associated with COVID-19: mortality attributable to COVID-19, instances of cardiopulmonary resuscitation, the requirement for mechanical ventilation, renal replacement therapy, extracorporeal membrane oxygenation, and admission to an intensive care unit for critical care. UDCA, ursodeoxycholic acid; CI, confidence interval.

Compared to controls, UDCA treatment had significant association with lower infection rate (OR, 0.69; 95% CI, 0.55–0.87) (Figure 2a). We performed sensitivity analysis, excluding Lee et al. (2024) (open-ended category) (Supplementary Figure 1). Compared to controls, UDCA treatment had significant association with lower severe infection risk (OR, 0.75; 95% CI, 0.64–0.89) and ventilator use (OR, 0.75; 95% CI, 0.62–0.90) (Figures 2b–d). However, there was no significant difference in mortality rate between the UDCA group and controls (OR, 1.06; 95% CI, 0.90–1.25) (Figure 2). No significant differences were observed for outcome measures of recovery time, hospitalization, and ICU hospitalization (Supplementary Figure 2).

### Dose–response association

Regression analysis showed a statistically significant association of lower risk of infection rates and increased UDCA dose. The decrease in the infection rate was found with increasing dosage—OR, 0.940 when 150 mg of UDCA; OR, 0.625 when 300 mg of UDCA; OR, 0.910 when 450 mg of UDCA; OR, 0.445 when 750 mg of UDCA (Table 2).

**TABLE 2:** Moderator effects by covariates using meta-regression analysis.

Variable	Infection rate						Mortality rate					
	*k*	*β*	OR	95% CI		*p*	*k*	*β*	OR	95% CI		*p*
No. of total patients	10	0.000	​	0.000	0.000	0.004	6	0.000	​	0.000	0.000	0.813
Age	10	0.000	​	−0.015	0.014	0.961	6	0.010	​	−0.048	0.068	0.736
Female rate	10	0.051	​	−1.140	1.241	0.933	6	1.399	​	−0.845	3.644	0.222
COVID-19 vaccination rate	10	0.951	​	−0.712	2.614	0.262	5	−0.381	​	−1.505	0.743	0.507
UDCA dose	​	​	​	​	​	0.036	​	​	​	​	​	0.282
150 mg	1	​	0.940	0.910	0.970	​	​	​	​	​	​	​
300 mg	3	​	0.625	0.419	0.932	​	1	​	0.580	0.219	1.535	​
450 mg	1	​	0.910	0.866	0.956	​	1^a^	​	1.000	0.827	1.535	​
750 mg	3	​	0.445	0.208	0.951	​	​	​	​	​	​	​
Country	​	​	​	​	​	0.001	​	​	​	​	​	0.188
USA	1	​	0.520	0.388	0.697	​	1	​	0.580	0.219	1.535	​
China	5	​	0.530	0.347	0.808	​	1	​	0.669	0.013	33.779	​
Korea	3	​	0.883	0.802	0.971	​	​	​	​	​	​	​
Japan	1	​	1.094	0.477	2.512	​	1	​	1.778	1.071	2.952	​
Italy	3	​	0.883	0.802	0.971	​	2	​	1.011	0.849	1.204	​
France	1	​	1.094	0.477	2.512	​	1	​	1.542	0.439	5.417	​
Disease type	​	​	​	​	​	0.001	​	​	​	​	​	0.966
Chronic liver disease	4	​	0.591	0.432	0.809	​	5	​	1.059	0.890	1.260	​
Liver transplantation	1	​	0.523	0.345	0.793	​	​	​	​	​	​	​
Others	5	​	0.930	0.902	0.958	​	1	​	1.070	0.694	1.650	​
Treatment duration	​	​	​	​	​	0.894	​	​	​	​	​	-
Over 3 months	2	​	0.560	0.451	0.695	​	​	​	​	​	​	​
Over 1 month	3	​	0.461	0.185	1.147	​	​	​	​	​	​	​
Under 1 month	1	​	0.523	0.345	0.793	​	​	​	​	​	​	​
Statistical adjust	​	​	​	​	​	0.641	​	​	​	​	​	0.312
PSM	8	​	0.663	0.499	0.880	​	3	​	1.217	0.891	1.663	​
Crude	2	​	0.764	0.451	1.295	​	3	​	1.009	0.836	1.218	​
Quality assessment	​	​	​	​	​	0.532	​	​	​	​	​	0.646
Low risk of bias	5	​	0.751	0.595	0.948	​	3	​	1.151	0.677	1.956	​
Moderate risk of bias	3	​	0.446	0.183	1.087	​	​	​	​	​	​	​
Serious risk of bias	2	​	0.764	0.451	1.295	​	3	​	1.009	0.836	1.218	​

UDCA, ursodeoxycholic acid; *k*, number of effect sizes; *β*, regression coefficient; OR, odds ratio; *p*-value from meta-regression analysis using restricted maximum likelihood.

^a^ Colapietro et al. (2023) was calculated as 582 mg UDCA dose (9.7 mg/kg daily*average 60 kg). Lee et al. (2024) was calculated as 150 mg and 450 mg, respectively corresponding to the median for two groups with dose ranges of less than 300 mg and greater than 300 mg.

### Moderator analysis

The study also considered the potential moderating roles of the following variables using meta-regression analysis (Table 2). We did not find any potential moderator in continuous covariates. Among the categorical covariates, the difference in the risk of infection rate according to the dose in the UDCA group was statistically significant (*p* = 0.036). In addition, the risk of infection rate by disease type and country was statistically significant (p < 0.05 for each).

### Publication bias

The statistical approaches for the detection of publication bias or a small-study effect for each outcome are shown in Supplementary Figure 3. It is widely spread from side to side but shows symmetry, so there was no significant publication bias. Ventilator use, hospitalization, ICU hospitalization, and recovery time, which had a small number of studies, were excluded from the publication bias analysis, and all did not show publication bias in Egger’s regression test and the Begg and Mazumdar rank test (*p* > 0.05 for each).

### Quality assessment

A total of 15 studies were evaluated with ROBINS-I using seven domains to determine the risk of bias. In D1 and D2, two studies showed “moderate” and “serious” bias. In D3 and D6, all studies ranked “low” bias. In D4, seven studies were “moderate” due to deviations from intended treatment. Overall, seven studies (Lee et al., 2024; Hu et al., 2024; Okushin et al., 2025; Hu et al., 2025; Moon et al., 2024; Marrone et al., 2024; John et al., 2023a) were evaluated as having low bias, four (Yu et al., 2024; Okushin et al., 2024; Li D. et al., 2024; Li et al., 2023) as having moderate bias, and four (Li M. et al., 2024; Corpechot et al., 2024; Liu and Wang, 2023; Colapietro et al., 2023) as having serious bias (Supplementary Figure 4). We performed the sensitivity analyses excluding the serious risk-of-bias studies (Supplementary Figure 5).

## Discussion

To our knowledge, this is the first meta-analysis examining the clinical effects of UDCA in COVID-19 infection. Our systematic review and meta-analysis using 15 observational cohort studies with 716,310 participants (control 495,276 vs. UDCA 221,034) revealed that UDCA treatment was associated with decreased risk of infection rate (OR, 0.69), severe infection (OR, 0.75), and ventilator use (OR, 0.75) compared to controls that did not use UDCA. Moreover, there was a significant association with lower risks according to increased UDCA dose in dose–response meta-regression: 150 mg (OR, 0.82), 300 mg (OR, 0.67), and 750 mg (OR, 0.36). However, there was no significant difference in recovery time, hospitalization, ICU hospitalization, and mortality rates between control and UDCA.

### Strengths and limitations

This is the first systematic and dose–response meta-analysis to identify clinical effects of UDCA in COVID-19 infection. UDCA showed association of lower risk of COVID-19 infection to dose–response and lower risk of severe infection and use of ventilator and symptoms relief. Therefore, we cautiously suggest that UDCA could be administrated to prevent COVID-19 infection with safety and good tolerability, compared to monoclonal antibodies, in any new outbreak.

Nevertheless, our study has some limitations. First, included studies were from observational studies with substantial risk of bias. Second, there was a lack of patient-level data limited dose–response precision. There was a chance of misclassification when assigning the median dose in the case of open-ended categories. However, in such a case, the most reasonable method would be to use the median dose assignment (Shim and Lee, 2019; Shim et al., 2021; Xue et al., 2014). We performed sensitivity analysis. We divided studies as low dose (<300 mg) vs. high dose (>300 mg) and compared the results with or without Lee et al. (2024). We could not analyze for low dose because there was only one study. On the other hand, there were seven studies with high dose, so we used each actual dose for six studies except Lee et al. (2024). In seven studies including Lee et al. (2024), OR was 0.623 (95% CI, 0.453–0.857), and in six excluding Lee et al. (2024), OR was 0.568 (95% CI, 0.399–0.810). There was no statistically significant difference between them (p = 0.704). Therefore, for open-end categories, median dose assignment might appear to be misclassification, but it might be reasonable because of no statistical significance. Third, the study population was predominantly patients with chronic liver disease, limiting generalizability. Therefore, our results focused on potential high-risk, vulnerable populations, so it might be difficult to apply the effectiveness of UDCA to healthy individuals. However, considering the safety of UDCA and risk and impact of COVID-19, it could an option for prophylaxis and symptom relief. Nevertheless, there is an urgent need for RCT in the general population. Fourth, there were incomplete data on vaccination status and variants—key confounders. Detailed vaccination history (number of vaccinations and antibody formation after vaccination) was insufficient. However, most of the study provided information on approximate vaccination status during the COVID-19 pandemic. There was also an insufficient amount of covariate information, so we performed a meta-regression analysis to evaluate the potential moderating effects of various covariates, although we were not able to confirm the sufficient moderating effects. A fifth weakness of our study was residual confounding despite PSM in some studies. Among 15 studies, four showed a serious risk of bias. Nevertheless, eight studies were performed using a PSM approach, and most studies were well-balanced in terms of minimizing potential confounding factors. Finally, UDCA had no impact on hospitalization, ICU admission, and mortality, even though it lowered the risk of infection and severe infection. This means that the implications in terms of public health might not be significant in real-world practice.

Early in the pandemic, SARS-CoV-2 entered host cells through ACE2. Since then, researchers have been looking into ways to prevent COVID-19 infection by targeting ACE2 (Xu et al., 2021; Rodal et al., 2021). Brevini et al. (2023) found that FXR participated in the regulation of ACE2 expression in several tissues involved in SARS-CoV-2 replication. FXR is a ubiquitous bile-acid-sensing protein, and with its activation by chenodeoxycholic acid, the expression of ACE increased in biliary, airway, and intestinal organoids. FXR directly bound the ACE2 promoter and activated its transcription. Suppressing FXR activity using UDCA downregulated ACE2 expression and reduced SARS-CoV-2 infection *in vitro*, *in vivo*, and *ex vivo*. Moreover, UDCA treatment decreased infection when lungs were exposed to SARS-CoV-2. Similarly in our study, UDCA treatment was associated with decreased risk of infection rate (OR, 0.69) compared to non-UDCA treatment. In addition, the risk of infection gradually decreased according to dose increase (150 mg, 300 mg, and 750 mg: OR, 0.82, 0.67, and 0.36, respectively).

Excessive inflammatory reactions caused by SARS-CoV-2 infection were the main cause of severe infection (Vabret et al., 2020). UDCA’s immunomodulatory effects reduced the exacerbated immunologic response that occurred in autoimmune cholestatic diseases by inhibiting the upregulation of major histocompatibility complex antigens and perhaps by reducing the release of cytokines by immunocompetent cells (Roma et al., 2011). In our study, UDCA treatment decreased the risk of severe infection (OR, 0.75) and ventilator use (OR, 0.75) compared to controls, consistent with Brevini et al. (2023).

Even though vaccination is the only prophylactic strategy among the general population to prevent and attenuate COVID-19 infection through inducing immune-reaction and the formation of antibodies for virus, these effects are reduced by the emergence of vaccine-resistant virus variants (John et al., 2021; John et al., 2023b). Moreover, the neutralization effect was different among individuals (less than immunocompromised patients), and the efficacy of vaccine and neutralizing antibodies decreased over time (John et al., 2022; Ferreira and John, 2023). Treatments such as remdesivir and nirmatrelvir/ritonavir had various and significant side effects, including hepatotoxicity, and were limited for use in the general population (Wong et al., 2023; Beigel et al., 2020). Monoclonal antibodies such as tixagevimab, cilgavimab, and bebtelovimab faced challenges of high cost, intravenous administration and reduced efficacy against newer variants (John et al., 2023a; Wong et al., 2023). However, UDCA was less affected by virus variants and less susceptible to immune escape by viruses because, to prevent COVID-19 infection, UDCA targets the ACE2 receptor—the host cell protein rather than virus.

Compared to vaccines and monoclonal antibodies, UDCA has multiple advantages, including minimal side effects, fewer drug–drug interactions, easy oral administration, affordable costs, and/or its accessibility to health systems worldwide (Huang et al., 2023; Walker et al., 2020; Shi et al., 2006; Braga et al., 2009). Furthermore, UDCA has already been administered for extended periods for various clinical indications to susceptible populations that would benefit from chemoprophylaxis, such as liver or bone marrow transplants (Mohty et al., 2020; Hirschfield et al., 2017). It has a favorable safety profile and tolerability in susceptible populations, and the feasibility of UDCA as pharmacological prophylaxis against COVID-19 in these populations has been demonstrated. However, considering that vaccines had a vastly stronger evidence base for efficacy and public health impact in the general population, UDCA could be a potential complement in specific scenarios, although not a replacement of vaccines.

It might be difficult to generalize the taking of UDCA because most studies focused on chronic liver disease. However, it is worth considering the use of UDCA in with low- and middle-income countries where access to vaccines or monoclonal antibodies is difficult. Brevini et al. (2023) revealed that UDCA decreased the level of ACE2 in the nasal epithelium of healthy individuals and suggested a correlation between UDCA and favorable clinical outcomes in COVID-19 patients. In addition, since the prognosis of COVID-19 infection in patients with chronic liver disease was worse than in general, the use of UDCA might also be considered in developed countries.

Our dose–response meta-analysis revealed that the association of lower susceptibility to SARS-CoV-2 infection increased as UDCA dose increased. Brevini et al. (2023) showed significantly reduced ACE2 in nasal epithelial cells from the first day of 15 mg/kg/day administration and recovery to pre-administration level approximately 2 weeks after taking UDCA. In an acute hepatitis clinical setting, it took approximately 2 weeks to improve liver function in patients with UDCA (Kumar and Tandon, 2001). Considering these results based on observational data, we might carefully recommend a dose of UDCA between 300 mg and 750 mg over 2 weeks for lower COVID-19 infection risk. However, further RCT studies are needed for this recommended dosage.

## Conclusion

UDCA treatment was linked to a reduction in the rates of infection and severe infection of COVID-19 and ventilator use in patients with COVID-19 infection compared to patients without UDCA. Moreover, there was dose-dependent association of lower infection rates. Therefore, if COVID-19 infection occurred in patients taking UDCA, it would be helpful to maintain the administration of UDCA. Furthermore, considering the benefits of UDCA (e.g., accessibility, affordability, safety profile, and tolerability during long periods despite high dosage), UDCA could be considered for high-risk populations for its association with lower COVID-19-infection risk and symptoms. A well-designed prospective study is needed for application to the general population.

## Data Availability

The original contributions presented in the study are included in the article/Supplementary Material; further inquiries can be directed to the corresponding authors.
