# Supplementary material for: Clinical effects of ursodeoxycholic acid in COVID-19 infection: a systematic review and dose–response meta-analysis
Source: Front Pharmacol. 2026 Apr 1;17:1719144. doi: 10.3389/fphar.2026.1719144 (PMC13079291; doi:10.3389/fphar.2026.1719144)
Supplement: Supplementary file 1 [file Supplementaryfile1.docx]

**Supplementary appendix**

Preventive and clinical effects of ursodeoxycholic acid in COVID-19 infection: A systematic review and dose-response meta-analysis

This supplemental material has been provided by the authors to give readers additional information about their work.

eFigure 1. Forest plots for infection rate in a) all included studies, and b) specific dose studies.

eFigure 2. Forest plots for ICU hospitalization, Hospitalization, and recovery time. Note: UDCA, ursodeoxycholic acid; CI, confidence interval.

eFigure 3. Funnel plots for publication bias.

eFigure 4. Quality assessment using ROBINS-I.

eFigure 5.

eTable 1. Search queries.

eTable 2. Detailed characteristics of the included studies

eFigure 1. Forest plots for infection rate in a) all included studies, and b) specific dose studies.


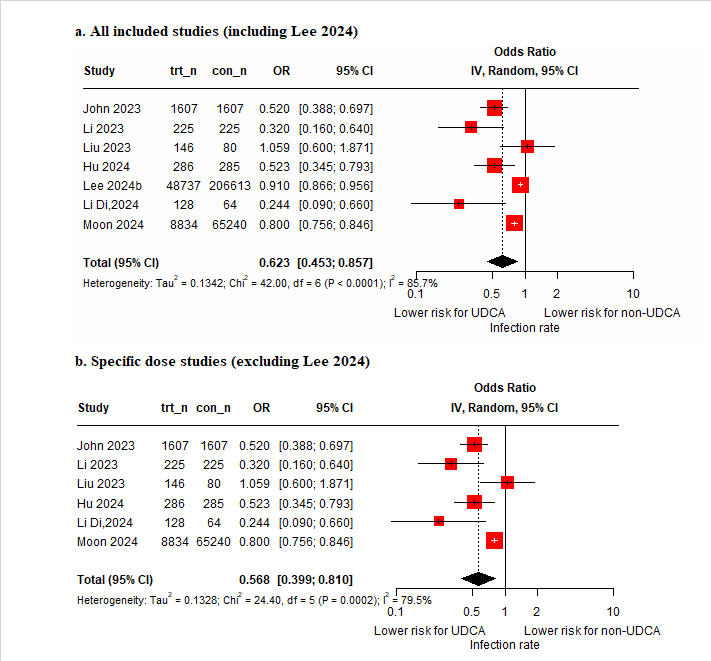


Only studies involving 300mg or more were analyzed for infection rates.

A comparison of studies that included or excluded Lee 2024 showed no statistically significant difference (p = 0.704).

eFigure 2. Forest plots for ICU hospitalization, Hospitalization, and recovery time.


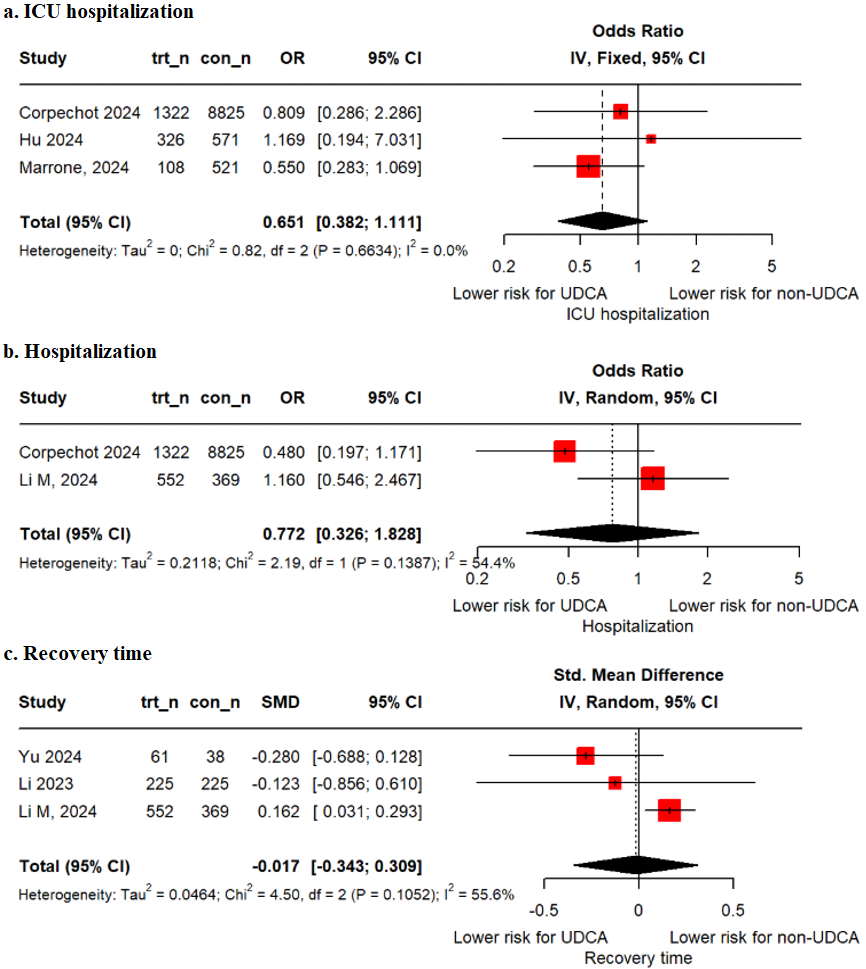


Note: UDCA, ursodeoxycholic acid; SMD, standardized mean difference; CI, confidence interval.

eFigure 3. Funnel plots for publication bias.


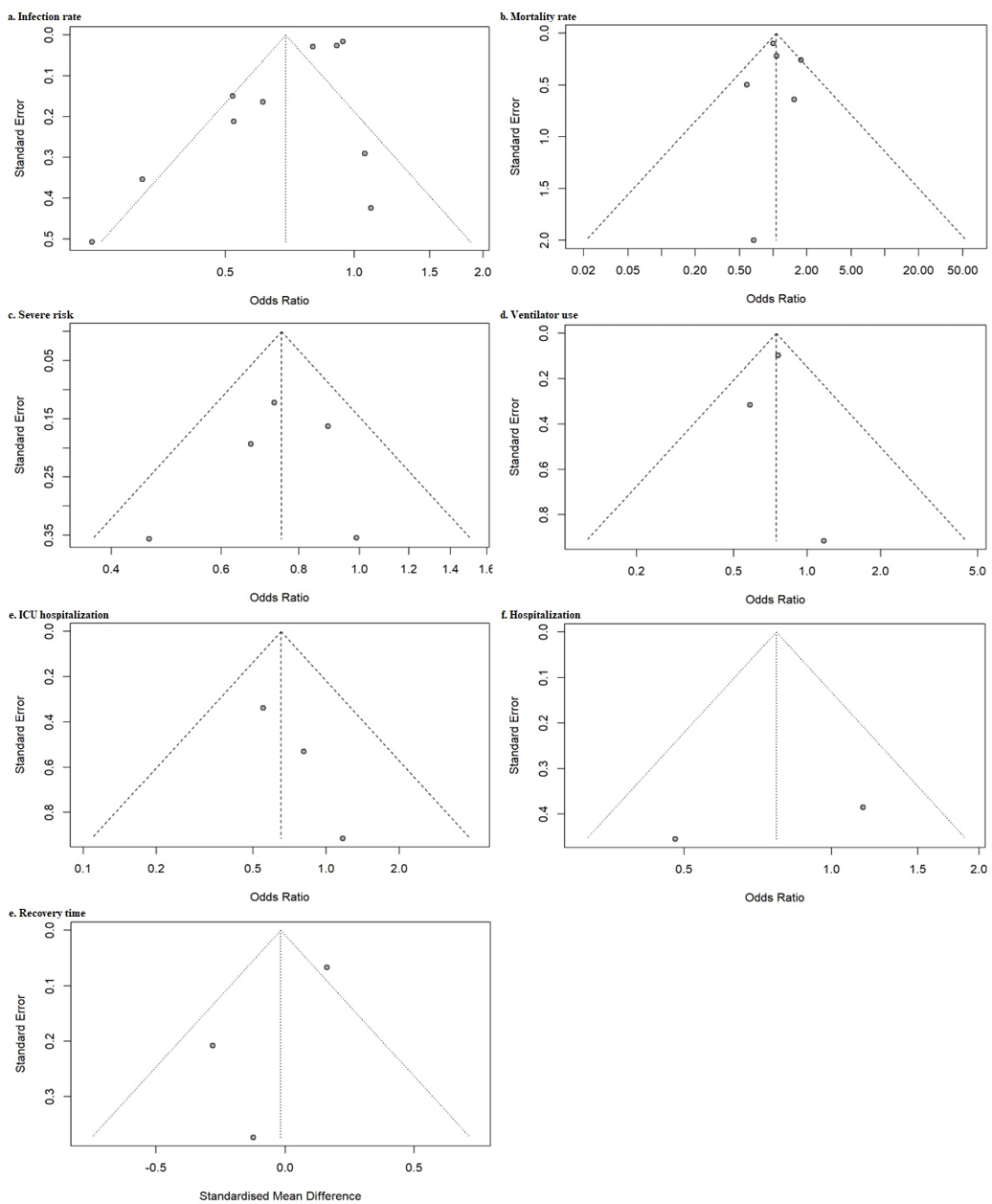


eFigure 4. Quality assessment using ROBINS-I.


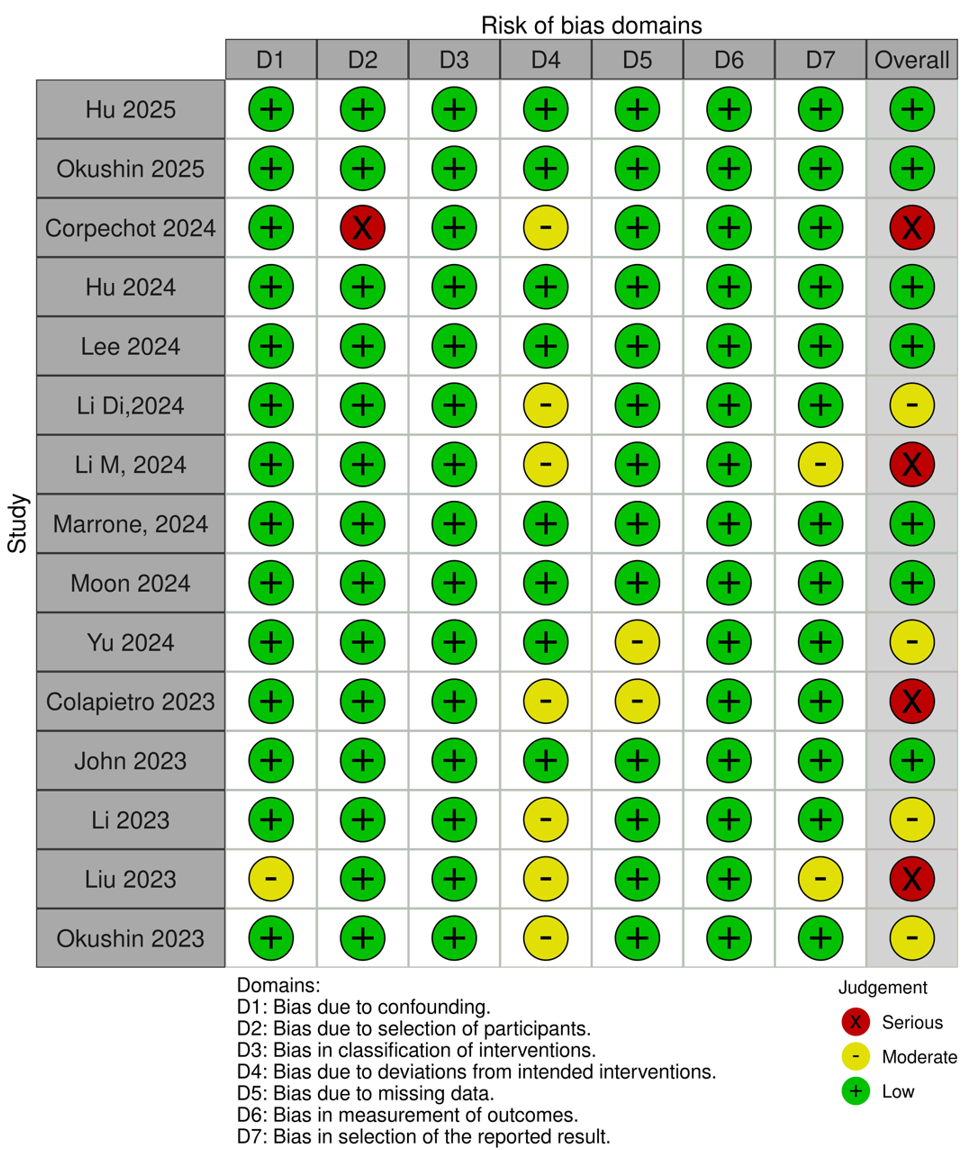


eFigure 5. Forest plots for a) infection rate, b) mortality rate, c) severe risk, and d) ventilator use excluding ‘serious’ risk-of-bias studies.


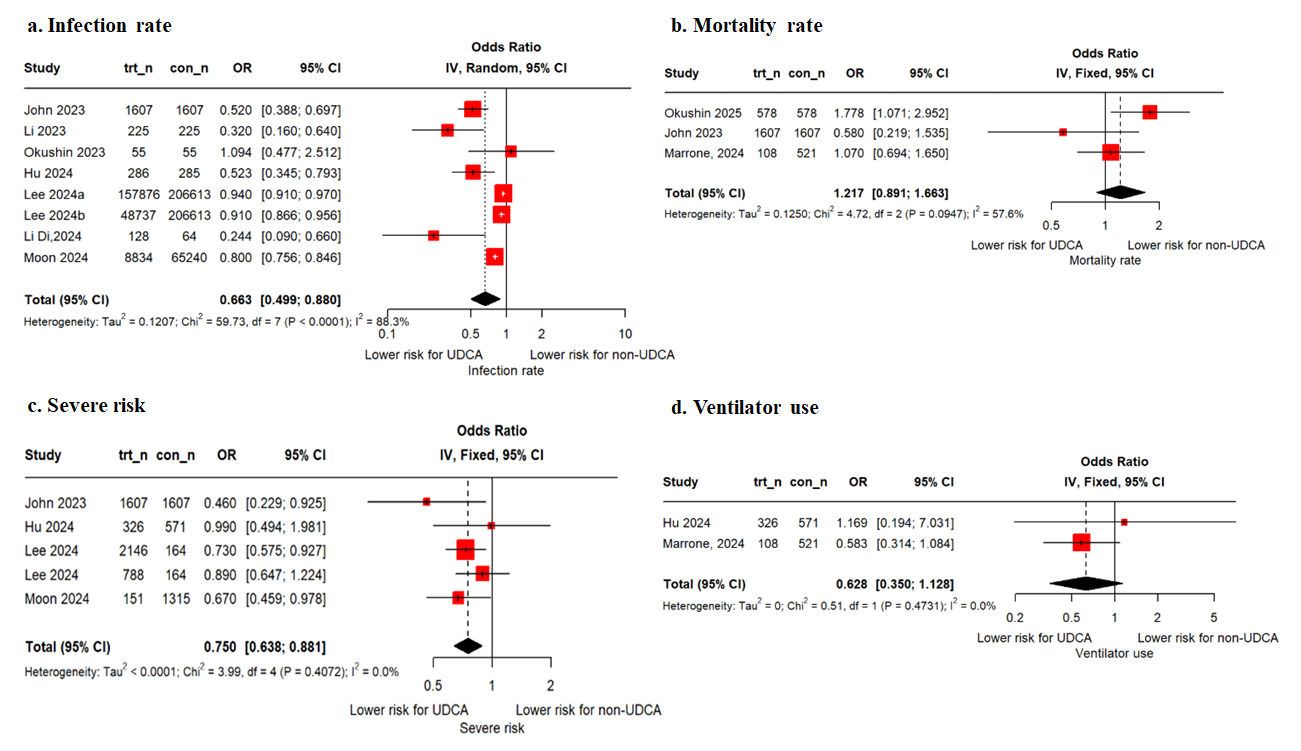


eTable 1. Search queries

| **PubMed and Cochrane Library** | | | | | | |
| --- | --- | --- | --- | --- | --- | --- |
|  | 1. Disease ("COVID-19"[Mesh] OR "SARS-CoV-2"[Mesh] OR "SARS-CoV-2 variants" [Supplementary Concept] OR "COVID-19 breakthrough infections" [Supplementary Concept] OR "COVID-19"[tiab] OR "COVID 19"[tiab] OR "COVID19"[tiab]) | | | | | |
|  | 2. Intervention ("Ursodeoxycholic Acid"[Mesh] OR "Ursodeoxycholic Acid"[tiab] OR "UDCA"[tiab] OR "Ursodiol" [tiab]) | | | | | |
|  |  |  |  |  |  |  |
| **Embase** | | | | | | |
|  | 1. Disease ('coronavirus disease 2019'/exp OR 'Severe acute respiratory syndrome coronavirus 2'/exp OR 'COVID-19':ti,ab OR 'COVID 19':ti,ab OR 'COVID19':ti,ab) | | | | | |
|  | 2. Intervention ('Ursodeoxycholic Acid'/exp OR 'Ursodeoxycholic Acid':ti,ab OR 'UDCA':ti,ab OR 'Ursodiol':ti,ab) | | | | | |

| **eTable 2. Detailed characteristics of the included studies** | | | | | | | | | | | |
| --- | --- | --- | --- | --- | --- | --- | --- | --- | --- | --- | --- |
|  | | | | | | | | | | | |
| Study | Age | Female rate | Country | Study design | Population | Intervention | Control | Outcome | UDCA treatment duration | COVID-19 vaccination rate | COVID-19 diagnosis criteria |
| Hu 2025† | 62 | 71.52% UDCA, 71.52% Non-UDCA | China | Retrospective case-control, single center by age and sex matching (December 7, 2022 to January 23, 2023) | COVID-19 in patients with chronic liver diseases (309 for UDCA, 309 for non-UDCA) | UDCA | Non-UDCA | asymptomatic infection rate | - | 32% for UDCA and 33.32% for Non-UDCA | RDTs and NAATs |
| Okushin 2025 | 73 | 45.8% UDCA, 45.5% Non-UDCA | Japan | Retrospective case-control by PSM (February 2020 to December 2022) | COVID-19 in patients with chronic hepatitis B or C (579 for UDCA, 5834 for non-UDCA) | UDCA | Non-UDCA | Mortality rate | Over 3 months | - | ICD-10 code, and data from Medical Data Vision Co., Ltd. |
| Corpechot 2024 | 56.7 for UDCA,  55.0 for Non-UDCA | 71.7% UDCA, 35.9% Non-UDCA | France | Retrospective cohort, multi center (March 1, 2020 to December 31, 2020) | PBC, PSC for UDCA vs. CHB, CHC for Non-UDCA (1322 for UDCA, 8825 for non-UDCA) | UDCA | Non-UDCA | Mortality rate, hospitalization, and ICU hospitalization | Over 3 months | 0% | ICD-10 code |
| Hu 2024‡ | 50 | 25.1 UDCA, 17.2% Non-UDCA | China | Retrospective cohort, multi center by PSM (January 2015 to December 2022) | Immunosuppressed recipients for liver transplantation (326 for UDCA, 571 for non-UDCA) | UDCA 300mg/day (5mg/kg daily*average 60kg) | Non-UDCA | Infection rate, severe risk, ICU hospitalization, and ventilator use | Over 2 weeks | 42.0% for UDCA and 47.7% for Non-UDCA | RT-PCR |
| Lee 2024⁰ | 70 for UDCA, 40 for Non-UDCA | 36.3% UDCA, 35.3% Non-UDCA | Korea | Retrospective cohort study using NHIS by PSM (from 2015 to 2021) | Nationwide cohort (220,005 for UDCA, 4,122,560 for non-UDCA) | UDCA 150mg, 450mg | Non-UDCA | Infection rate and severe risk | - | 49.8% for UDCA, 49.0% for Non-UDCA | ICD-10 |
| Li Di,2024 | 56 | 63.8% UDCA (73 matched UDCA, 36 for non UDCA) | China | Retrospective cohort, single center by PSM (July 2022 to December 2022) | Outpatient (1,040 for UDCA, 64 for non-UDCA) | UDCA 750mg/day (5-20mg/kg daily*average 60kg) | Non-UDCA | infection rate | Over 1 month | 38.3% for UDCA, 28.1% for Non-UDCA | RDTs and NAATs |
| Li M, 2024 | 57.94 | 83.14% UDCA, 68.75% Non-UDCA | China | Prospective study with investigative telephone survey (January 2022 to January 2023) | Autoimmune liver disease patients (PBC, AIH, PSC) (706 for UDCA, 432 for non-UDCA) | UDCA | Non-UDCA | Infection rate, mortality rate, recovery time, and hospitalization | Over 3 months | 62.89% for UDCA,73.61% for Non-UDCA | RDTs and clinical and imaging examinations |
| Marrone, 2024 | 69 | 41.0% UDCA, 40.9% Non-UDCA | Italy | Retrospective case-control, single center by PSM (March 2020 to December 2022) | COVID-19 in patients admitted to emergency department (109 for UDCA, 6,335 for non-UDCA). | UDCA | Non-UDCA | Mortality rate, ICU hospitalization, and ventilator use | - | 16.7% for UDCA, 19.8%Non-UDCA | data from EMR |
| Moon 2024 | 56.6 | 47.0% UDCA, 47.3% Non-UDCA | Korea | Retrospective cohort study using NHIS by PSM (January 2020 to December 2021) | Chronic liver disease (8,834 for UDCA, 65,240 for non-UDCA) | UDCA 750mg | Non-UDCA | Infection rate and severe risk | - | - | RT-PCR assays conducted  on nasopharyngeal or oropharyngeal swabs |
| Yu 2024 | 23 | 38.5% UDCA, 36% Non-UDCA | China | Retrospective case-control, single center (December 2022) with meta-analysis | COVID-19 in patients with no comorbidities (65 for UDCA, 50 for non-UDCA) | UDCA 150mg, 300mg | Non-UDCA | Body temperature recovery and recovery time | - | 100% | data from EMR |
| Colapietro 2023 | 74 | 45.6% UDCA, 39.4% Non-UDCA | Italy | Retrospective case-control, single center (January 2020 to January 2023) | COVID-19 in patients with cholelithiasis, cholestatic liver diseases, and bone marrow diseases (57 for UDCA, 3790 for non-UDCA) | UDCA 582mg/day (9.7mg/kg daily*average 60kg) | Non-UDCA | Mortality rate and ventilator use | Over 3 months | 54.4% for UDCA and vs. 30.2% for Non-UDCA | data from EMR |
| John 2023 | 62 | 7.1% UDCA, 5.4% Non-UDCA | USA | Retrospective cohort by PSM (January 2008 to December 2018, with follow-up until 11 February 2022) | Cirrhosis (88 for UDCA, 6523 for non-UDCA). | UDCA 300mg/day (5mg/kg daily*average 60kg) | Non-UDCA | Infection rate, mortality rate, and severe risk | Over 3 months | 57.2% for UDCA and vs. 53.3% for Non-UDCA (booster dose, 30.2% vs. 24.1%). | PCR, ICD-10 code, and data from Veterans Outcomes and Costs Associated with Liver (VOCAL) cohort |
| Li 2023 | 53.3 | 59.6% UDCA, 56.9% Non-UDCA | China | Retrospective cohort, single center by PSM (January 2022 to December 2022) | Patients (hepatitis B and autoimmune hepatitis) (329 for UDCA, 325 for non-UDCA). | UDCA 750mg/day | Non-UDCA | Infection rate and recovery time | Over 1 month | 44.4% for UDCA and 64.9% for Non-UDCA | ICD-10 code and data from EMR |
| Liu 2023 | 4.7 for UDCA; 6.7 for Non-UDCA | 41.1% UDCA, 43.75% Non-UDCA | China | A questionnaire-based study (December 2022 to January 2023) | Children from families and members (146 for UDCA, 80 for non-UDCA). Children's Hospital of Fudan University for liver disease in the preceding 5 years | UDCA 300mg/day (10-20mg/kg daily*average 20kg) | Non-UDCA | infection rate | Over 1 month | 44% for UDCA and 49.2% for Non-UDCA | survey |
| Okushin 2023 | 69 | 69.0% UDCA, 45.1% Non-UDCA | Japan | Retrospective case-control, single center by PSM | Outpatients. No complication information (55 for UDCA, 335 for non-UDCA). | UDCA | Non-UDCA | Infection rate | - | 90.91% for UDCA and 82.27% for Non-UDCA | medical interview and IgG-N positive |
| †Hu 2025 was used only in the quality assessment. ‡Hu 2024 used the risk of the entire dose when analyzing individual outcome, and the risk of individual dose groups from 300-1050 mg when performing DRMA analysis. ⁰Lee 2024 was calculated as 150mg and 450mg, respectively, corresponding to the median for two groups with dose ranges of less than 300mg and greater than 300mg  UDCA, ursodeoxycholic acid; RDT, rapid antigen diagnostic tests; NAAT, nucleic acid amplification tests; PSM, propensity score matching; PBC, primary biliary cholangitis; PSC, primary sclerosing cholangitis; CHB, chronic hepatitis B; CHC, chronic hepatitis C; ICU, intensive care unit; ICD-10, international statistical classification of diseases and related health problems 10^th^ revision;  RT-PCR, real time-polymerase chain reaction, NHIS, national health insurance service; AIH, autoimmune hepatitis. | | | | | | | | | | | |
